# Phytochemical Analysis and Neuroprotective Effect of *Salvia castanea* Diels f. *Tomentosa* Stib Extracts

**DOI:** 10.3390/ph18050728

**Published:** 2025-05-15

**Authors:** Xiaoyan Peng, Yuxing Dai, Jianwen Chen, Jing Lu, Dan Zhou, Fahuan Ge, Peiqing Liu, Xue Zhou

**Affiliations:** 1 Department of Pharmacognosy and Natural Medicinal Chemistry, School of Pharmaceutical Sciences, Sun Yat-Sen University, Guangzhou 510006, China; pengxy83@mail.sysu.edu.cn (X.P.); gefahuan@mail.sysu.edu.cn (F.G.); 2 Guangdong Research Center for Supercritical Fluid Extraction of Chinese Medicine, Guangzhou 511458, China; 3 National and Local United Engineering Laboratory of Druggability and New Drugs Evaluation, School of Pharmaceutical Sciences, Sun Yat-Sen University, Guangzhou 510006, China; daiyx5@mail2.sysu.edu.cn (Y.D.); chenjwen@mail.sysu.edu.cn (J.C.); lujing28@mail.sysu.edu.cn (J.L.); zhoudan7@mail.sysu.edu.cn (D.Z.); 4 Guangdong Provincial Key Laboratory of New Drug Design and Evaluation, School of Pharmaceutical Sciences, Sun Yat-Sen University, Guangzhou 510006, China

**Keywords:** *Salvia castanea* Diels f. *Tomentosa* Stib, total tanshinones, total phenolic acids, process optimization, chemical composition, neuroprotective effect, ischemic stroke

## Abstract

**Background**: Early studies indicated that the high content of tanshinone IIA (T−IIA) and rosmarinic acid (RA) in *Salvia castanea* Diels f. *Tomentosa* Stib (SCT) gives them significant potential for development as therapeutic agents for ischemic stroke (IS). However, the extraction process and quality of the active ingredients from SCT are still big challenges, with present processes providing insufficient pharmacological effects. This study aims to identify the optimal extraction process and perform a quality characterization of the total tanshinones and phenolic acids extracted from SCT, as well as to elucidate the neuroprotective effect of these extracts. **Methods**: The extraction process was optimized using an orthogonal experimental design (OED), and quality characterization was performed using HPLC, UV, and LC-MS. The neuroprotective effect of the extracted tanshinones and phenolic acids was studied using the middle cerebral artery occlusion (MCAO) paradigm, and its underlying mechanism was revealed through RNA-seq analysis combined with network pharmacology. **Results**: The optimal extraction pressure of total tanshinones was 60 MPa, while the extraction temperature and time for total phenolic acids were 4 °C and 25 min, respectively. In these extracts, the total tanshinone and phenolic acid contents increased to 369.43 and 189.10 mg/g, respectively; 23 of the 19 tanshinones and 23 phenolic acids identified in this study have not been observed in previous studies. It was demonstrated that the combined extract had a promising neuroprotective effect against IS; RNA-seq combined with network pharmacology analysis indicated that the active compounds may regulate a series of core genes associated with signaling pathways to protect against IS. **Conclusions**: The combined SCT extract studied in this research exerted neuroprotective effects on IS. In general, these findings improve our preliminary understanding of the chemical composition and bioactivity of SCT.

## 1. Introduction

Stroke is the second leading cause of mortality worldwide [1], representing a significant public health concern. It is an acute cerebrovascular disease that is a prevalent cause of mortality among the elderly [2,3,4]. IS is the most common kind of stroke, accounting for approximately 87% of all cases [5]. The principal therapeutic approaches for IS are intravenous thrombolysis [6] and vascular intervention [7]. However, many deficiencies limit treatment prognosis, including a restricted therapeutic time window [8], advanced patient age, genetic predispositions, environmental influences, etc.

Furthermore, the management and outcomes of ischemic stroke are influenced by various complex factors. Recent research has highlighted challenges in ensuring timely access to treatment, which can be particularly problematic during public health crises, significantly impacting patient prognosis [9]. Additionally, identifying reliable biomarkers and understanding factors, such as the blood urea nitrogen-to-albumin ratio, that correlate with long-term clinical outcomes remain active areas of investigation, underscoring the intricate nature of stroke recovery [10]. Consequently, there remains a critical need for exploring and developing novel therapeutic strategies. A major therapeutic goal following ischemic stroke is neuroprotection—strategies aimed at salvaging brain tissue within the ischemic penumbra by interfering with the pathophysiological cascade leading to delayed neuronal death. Preclinical studies often compare the efficacy of different neuroprotective compounds, such as dl-3-n-butylphthalide and edaravone dexborneol in rat models of cerebral ischemia, seeking agents with superior protective effects [11]. Natural products, with their diverse chemical structures and potential for multi-target effects, represent a promising source for discovering novel neuroprotective candidate compounds. Among these, species from the Salvia genus have garnered significant attention.

*Salvia miltiorrhiza* Bunge (SMB), a traditional Chinese medicinal plant, has shown beneficial effects in the treatment of stroke in numerous clinical trials [12]. Recently, several studies focused on the effect of single chemical components, such as tanshinone or phenolic acid extracted from SMB, on IS [13,14]. They found that T−IIA from tanshinones and RA from phenolic acids are important neuroprotectors [15,16,17]. The trend toward multi-substance combination therapy for IS is on the rise [18]. However, there are few reports on the neuroprotective effect of the combined application of phenolic acids and tanshinones on IS.

SCT and SMB are both plants of the Salvia genus, mainly distributed in Nyingchi, Tibet, China [19]. SCT is often used as a substitute for SMB in Tibet. The constituents of SCT in terms of liposoluble tanshinones and water-soluble phenolic acids are similar to those of SMB [20]. However, SCT distinguishes itself through its content of specific components, notably its significantly higher levels of T−IIA and RA compared to SMB [21]. This characteristic confers upon it a notable advantage over its counterpart. From a biochemical and pharmacological perspective, this component content difference may indicate SCT’s unique potential and value in pharmacodynamics and clinical application. However, current studies on SCT mainly focus on the isolation and identification of chemical components [22], biosynthesis [23], and histological regulation [24]. Therefore, the question of whether SCT is more effective than SMB in the treatment or prevention of IS requires further verification.

Extraction is the first and most important process for assessing the quality of traditional Chinese medicinal materials, directly influencing the types and quantities of active ingredients derived from them, as well as the extent of their efficacy [25]. Currently, traditional solvent extraction is the primary extraction method for SCT. However, this approach presents several drawbacks, including the use of a singular extraction solvent, high temperatures, and prolonged extraction durations. Additionally, the extracted substance often contains solvent residues, presents a homogenous composition of active ingredients, and suffers from the degradation of thermally unstable components, leading to a reduction in their concentration and posing significant challenges to maximizing pharmacological activity [26]. Consequently, developing an efficient extraction method is imperative. Our team has extensively investigated high-pressure supercritical fluid extraction (HPSFE) and high-pressure disruption extraction (HPDE). The findings reveal that HPSFE under elevated pressure significantly improves the extraction efficiency of liposoluble components. Moreover, both extraction techniques demonstrate distinct advantages in extracting liposoluble and thermally sensitive compounds [27,28,29]. Therefore, combining HPSFE and HPDE for SCT extraction may enhance both the concentration and therapeutic efficacy of its active ingredients.

IS initiates a cascade of pathogenic events, including excitotoxicity, oxidative stress, neuroinflammation, and dysregulated lipid metabolism, ultimately leading to neuronal apoptosis and persistent neurological dysfunction. Current therapeutic approaches primarily target these interconnected pathways. Notably, emerging evidence underscores lipid metabolism dysregulation as a pivotal contributor to both ischemic pathogenesis and post-stroke functional outcomes, particularly in cognitive recovery. Targeting lipid metabolism has emerged as a potential therapeutic avenue. For instance, natural compounds like Mangiferin have demonstrated beneficial effects, such as alleviating post-stroke cognitive impairment [30]. Moreover, key pathways involved in cell survival and tissue repair following ischemia include angiogenesis and anti-apoptotic signaling. Vascular Endothelial Growth Factor (VEGF) is a crucial mediator of angiogenesis and neurogenesis, playing a protective role by promoting blood vessel formation and neuronal survival in the ischemic penumbra [31]. Furthermore, the Phosphatidylinositol 3-kinase/Akt (PI3K/Akt) signaling pathway is a central regulator of cell survival, proliferation, and metabolism; its activation, often measured by the phosphorylation of Akt (p-Akt), is known to inhibit apoptosis and promote recovery after cerebral ischemia [32,33]. Given the multi-target potential of herbal extracts, investigating the effect of SCT extract on VEGF expression and Akt phosphorylation provides valuable insights into its putative neuroprotective mechanisms against IS.

To the best of our knowledge, no previous studies have explored the combined extraction and neuroprotective effects of SCT in IS treatment. In this study, we optimized SCT extraction methodologies to enhance the contents of total tanshinones and total phenolic acids and characterized the quality of their extracts. Furthermore, we demonstrated the neuroprotective effect of the combined application of total tanshinones and total phenolic acids extract in an MCAO model of IS. RNA-seq and network pharmacology analysis were used to investigate the preliminary mechanism of action of their combination.

## 2. Results and Discussion

### 2.1. Extraction Process Optimization of Total Tanshinones and Total Phenolic Acids

OED was used to evaluate the effects of different independent variables (each at three levels) on the extraction efficiency of total tanshinones and total phenolic acids from SCT. The composite scores and extreme variance analysis results are summarized in Table 1 and Table 2. As shown in Table 1, the key factors examined in HPSFE were the extraction pressure (MPa, A), extraction temperature (°C, B), entrainer multiplier (mL/g, C), and separation kettle I pressure (MPa, D). As we know, the larger the R value for a factor, the stronger the influence of the test factor on the result. The four factors with the greatest influence on the extraction of total tanshinones were found to be, in descending order of importance, the multiplicity of the entraining agent, followed by extraction temperature, separation kettle I pressure, and extraction pressure.

The ANOVA results (Table 3) revealed significant differences among all investigated factors. Through analysis of the k mean values, the optimal level for each extraction parameter was determined. Extreme variance analysis identified A_1_B_2_C_3_D_1_ as the optimal combination for total tanshinone extraction. Specifically, the optimal HPSFE conditions of total tanshinones were as follows: extraction pressure of 60 MPa, extraction temperature of 55 °C, entrainer multiplicity of 2.5 times, and separation I pressure of 6 MPa.

Similarly, this study primarily examined the effects of the material–liquid ratio (g/mL, A), extraction time (min, B), and extraction temperature (°C, C) on HPDE for total phenolic acids from the residual SCT material. As shown in Table 2, the material–liquid ratio and extraction time exhibited the strongest influence on phenolic acid yield, followed by the extraction temperature. The ANOVA results (Table 4) confirmed the significant effects of all factors. Consequently, based on the level factor results, the optimal levels for each single factor in the extraction of total phenolic acid from the remaining SCT residue should be A_3_B_3_C_1_. Specifically, the optimal HPDE conditions of total phenolic acids from the remaining residue of SCT after HPSFE were as follows: material–liquid ratio of 1:40, extraction temperature of 4 °C, and extraction time of 25 min.

In this study, total tanshinones and total phenolic acids were extracted from SCT by HPSFE coupled with HPDE for the first time. OED was employed to optimize the extraction parameters, yielding a stable and reproducible protocol. Compared with the extraction of SCT via supercritical CO_2_ at a pressure less than 40 MPa under conventional pressure, the high-pressure extraction we utilized can greatly improve efficiency [34]. Other studies have shown that compared with traditional extraction methods, high-pressure supercritical CO_2_ extraction has the characteristics of simpler and safer operation, higher extraction yields and energy efficiency, and a larger scale. These findings support the broader application of HPSFE in SCT processing. In addition, HPDE, which can generate instantaneous high pressure and pressure drops on the liquid vehicle at low temperatures and enhance the release of active ingredients, helps to shorten the extraction time and improve the extraction efficiency. It is suitable for the extraction of various bioactive ingredients, such as polyphenols, flavonoids, and polysaccharides [35]. Similarly, the optimal HPDE conditions of total phenolic acids from the remaining residue of SCT after HPSFE were an extraction temperature of 4 °C and an extraction time of 25 min, which can efficiently preserve heat-sensitive components.

**Figure 1:**
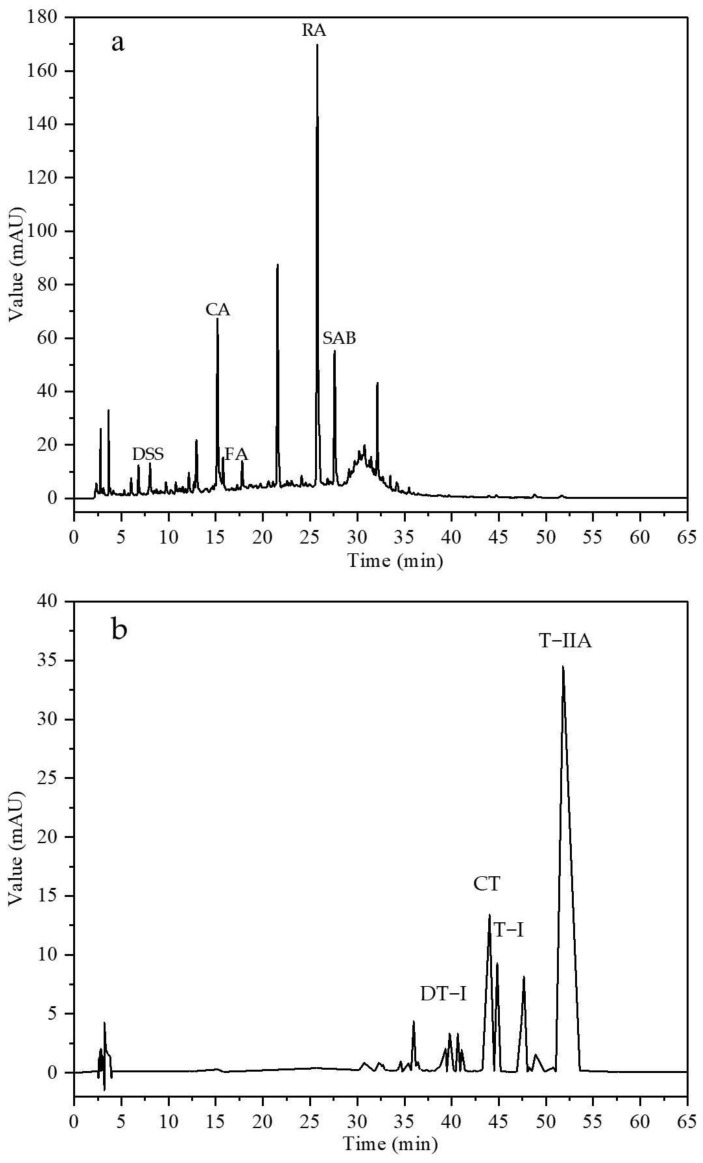
HPLC chromatograms of total phenolic acids and total tanshinones extract from SCT. (**a**) Total phenolic acid extract. (**b**) Total tanshinone extract.

Based on the established quantitative analysis methods, the key index components of total phenolic acids and total tanshinones extracts from SCT were determined under optimal extraction conditions, and high-performance liquid chromatography images were obtained (Figure 1). The results indicated that the total phenolic acid content in the extracts was approximately 189.10 mg/g. Specifically, the contents of danshensu sodium (DSS), caffeic acid (CA), ferulic acid (FA), RA, and salvianolic acid B (SAB) were 0.74, 1.93, 0.54, 21.26, and 4.54 mg/g, respectively (Table 5). Similarly, the total tanshinone content in the extracts was determined to be 369.43 mg/g, with the contents of dihydrotanshinone I (DT−I), cryptotanshinone (CT), tanshinone I (T−I), and T−IIA being 5.97, 39.13, 20.73, and 151.27 mg/g, respectively (Table 6).

An analysis of the extraction products revealed that RA among phenolic acids and T−IIA among tanshinones were the predominant constituents in SCT. The extraction yields were determined to be 3.12% for total tanshinones and 26.13% for total phenolic acids, with corresponding contents of 369.43 mg/g and 189.10 mg/g in final extracts, respectively. When compared with the contents of these components reported in the literature [36], the levels of RA, SAB, and four tanshinone components in SCT extracts during this study were significantly elevated. Most notably, when compared with CT and T−IIA extracted using conventional ultrasonic extraction methods [37,38], the contents of CT and T−IIA obtained using the extraction method adopted in this study increased by tens of times. These results clearly indicated that the extraction of target compounds from SCT was effective, and the employed extraction method was superior, leading to a higher concentration of active constituents in the samples. The substantial improvement in active component yield suggests these optimized extracts may offer greater therapeutic efficacy for disease treatment and prevention applications.

### 2.2. The Chemical Compositions of Total Tanshinones and Total Phenolic Acids Extracts

**Figure 2:**
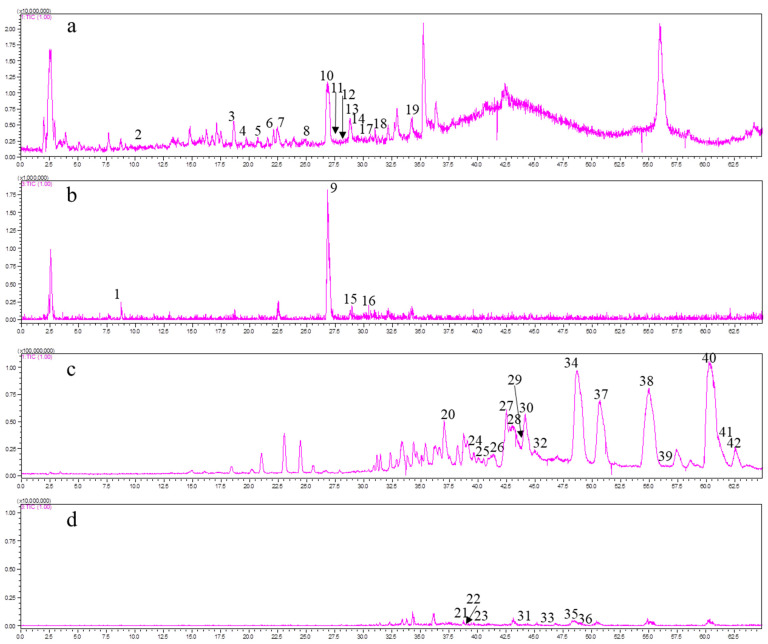
The total ion chromatograms of total tanshinone and total phenolic acid extracts from SCT. (**a**,**b**) Positive and negative ion modes of total phenolic acid extract. (**c**,**d**) Positive and negative ion modes of total tanshinone extract. The numbered peaks represent the positions of identified chemical components.

The SCT extracts were analyzed and qualitatively identified under established analytical conditions using high-resolution mass spectrometry. Compound identification was achieved through MS^n^ fragmentation cleavage ions, comparison with authentic reference standards, and literature data correlation. The total ion chromatograms for both phenolic acid and tanshinone fractions are shown in Figure 2. Comprehensive identification results, including retention times, molecular weights, and characteristic fragments, are summarized in Table 7 (total phenolic acid extract) and Table 8 (total tanshinone extract), respectively.

A total of 19 compounds were identified from the total phenolic acid extract, including 4 detected in the negative ion mode and the remaining 15 detected in the positive ion mode (Table 7). The identified components of the total phenolic acid extract comprised 11 phenolic acids, 6 flavonoids, 1 triterpenoid, and 1 other compound. Among these, five compounds matched reference substances: DSS (1), CA (6), RA (9), and SAB (14). Similarly, 23 compounds were identified from the total tanshinones extract, with 7 detected in the negative ion mode and the remaining 16 detected in the positive ion mode (Table 8). All 23 compounds in the total tanshinones extract were diterpenoids. Additionally, four components—DT−I (28), CT (34), T−I (37), and T−IIA (40)—were compared for validation.

Considering the identification results, there was a significant increase in 23 compounds compared to those reported in existing studies [39]. This also provides a more comprehensive reference for the pharmacodynamic material basis studies and quality control standardization of SCT. Additionally, based on the identified chemical components and their comparison with control products, it will be beneficial in determining key index components for quantitative detection. The experiment leveraged the high resolution and sensitivity of HPLC/MS-IT-TOF, enabling rapid and efficient separation and identification of compounds in complex mixtures. It identified the chemical components of total tanshinones and total phenolic acid extracts under its optimal conditions, aiming to fully utilize the active components and pharmacological effects of SCT. Furthermore, this study contributes to overcoming the current limitations in SCT material basic research. However, qualitative research on chemical components has a certain degree of subjectivity, and traditional Chinese medicine or natural products have the difficulty of multiple components and multiple targets. Therefore, all the chemical components in the samples have not been identified completely.

### 2.3. The Neuroprotective Effect of Total Tanshinone Combined with Total Phenolic Acid Extracts of SCT on IS

#### 2.3.1. SCT Reduces CIRI and Alleviates Neurological Deficits

**Figure 3:**
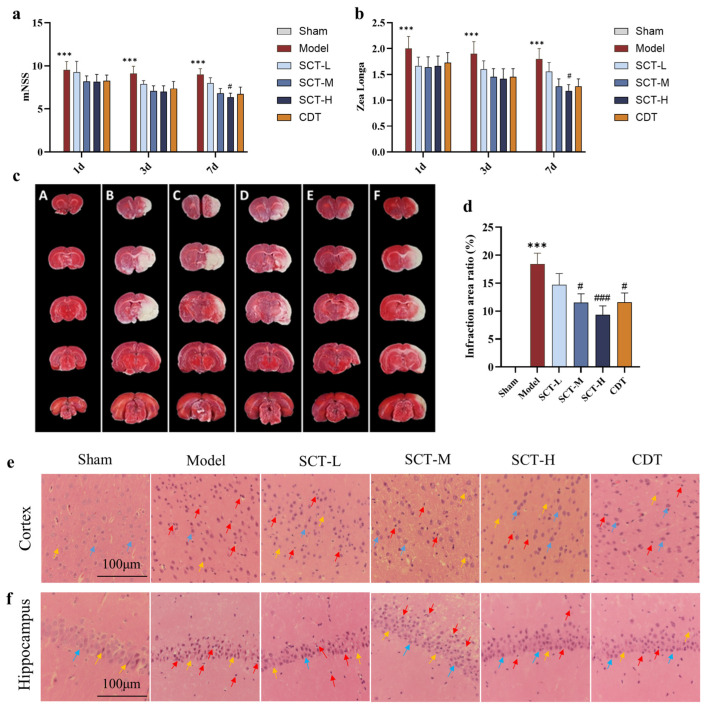
SCT reduces cerebral injury and alleviates neurological deficits after MCAO. (**a**,**b**) mNSS and Zea Longa were used for neurological functions and behaviors assessment after MCAO surgery *(n* = 9–12). (**c**,**d**) Brain infarct volume was assessed by TTC 7 days after MCAO (*n* = 9–12). Images legend: A (Sham), B (Model), C (SCT-L), D (SCT-M), E (SCT-H) and F (CDT). (**e**,**f**) HE staining was applied to evaluate the histopathological alterations of neuronal cells (*n* = 3). Scale bar = 100 μm. Data are mean ± SEM. ^#^ *p* < 0.05, ^###^ *p* < 0.001 vs. model group. *** *p* < 0.001 vs. sham group.

We applied Zea Longa and modified neurological severity score (mNSS) to assess the neurological function deficits and behavior changes post-MCAO after 1 day, 3 days, and 7 days. The results demonstrated that the animals in the model group had higher scores of both Zea Longa and mNSS compared with the sham group (*p* < 0.01). The animals treated with SCT showed lower scores relative to the model group, indicating the improvement in neurological deficits after MCAO (Figure 3a,b).

As the gold standard, 2,3,5-triphenyltetrazolium chloride (TTC) staining was used to evaluate brain infarct volume 7 days after MCAO in rats. Normally, viable brain regions stain red, while infarcted areas appear white. The results demonstrated that the infarct volume in the model group exhibited severe localized ischemia compared with the sham group, confirming the success of the MCAO model. Relative to the model group, SCT treatment groups (SCT-L, low-dose group of SCT; SCT-M, medium-dose group of SCT; SCT-H, high-dose group of SCT) and CDT (Compound Danshen Tablets), the positive control group, showed a reduction in the area of cerebral infarction to varying degrees (Figure 3c,d).

Moreover, brain tissue sections were stained with hematoxylin and eosin (HE) to investigate whether SCT rescues neuronal apoptosis induced by ischemia/reperfusion. Observations indicate that SCT rescues edema (yellow arrows), degeneration (blue arrows), and necrosis (red arrows) in the neuronal cells of the brain cortex and hippocampus compared with the model group (Figure 3e,f).

#### 2.3.2. SCT Exerts Neuroprotective Effect via Attenuating Oxidative Stress and Neuroinflammation

**Figure 4:**
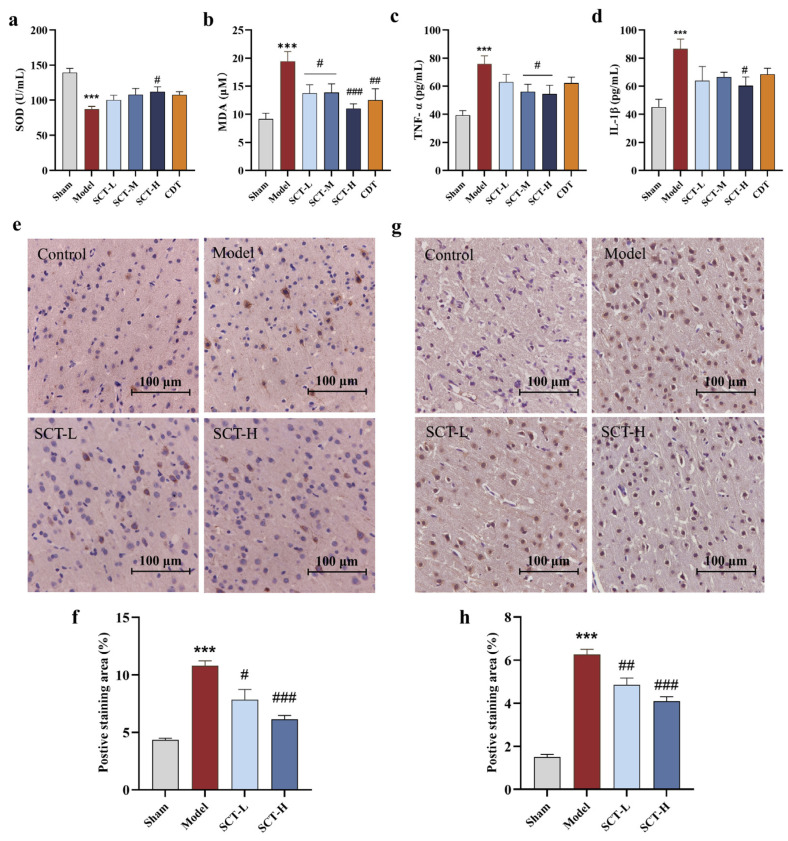
SCT attenuates oxidative stress and neuroinflammation. (**a**–**d**) SCT mediates the expression levels of SOD, MDA, IL-1β, and TNF-α in serum (*n* = 9–12). (**e**,**f**) Immunohistochemical staining was applied to investigate the expression levels of p-Akt (*n* = 3). (**g**,**h**) Immunohistochemical staining was applied to investigate the expression levels of VEGF-α (*n* = 3). Scale bar = 100 μm. Data are mean ± SEM. ^#^ *p* < 0.05, ^##^ *p* < 0.01, ^###^ *p* < 0.001 vs. model group. *** *p* < 0.001 vs. sham group.

Oxidative stress levels in serum were measured by malondialdehyde (MDA) and superoxide dismutase (SOD) levels. At 7 days after MCAO, MDA levels in the model group were significantly higher than the sham group, while the SOD level showed a marked decrease (*p* < 0.01). Compared with the model, SCT-H groups demonstrated a significant elevation in SOD levels (*p* < 0.05) and a reduction in MDA levels (*p* < 0.01) (Figure 4a,b). The levels of inflammatory cytokines, TNF-α, and IL-1β were determined to evaluate the anti-inflammatory effects of SCT. The TNF-αand IL-1β levels were significantly increased in the model group compared with the sham group (*p* < 0.01). Relative to the model group, the high-dose SCT group demonstrated a pronounced decrease in TNF-α (*p* < 0.05) and IL-1β (*p* < 0.01) (Figure 4c,d).

Given that VEGF-α plays a pivotal role in maintaining neurovascular unit homeostasis, and p-Akt serves as an indicative marker overseeing cellular apoptosis, immunohistochemical staining was employed to elucidate the expression levels in the sham, model, and both low- and high-SCT dosage groups. The staining outcomes revealed that MCAO precipitates a significant up-regulation of p-Akt (Figure 4e,f) and VEGF-α (Figure 4g,h) expression. Conversely, SCT was observed to inhibit their expression, thereby conferring a neuroprotective effect.

Furthermore, our IHC analysis focused on the effects of different doses of SCT compared to the model group to understand its specific impact on VEGF and p-Akt expression. While CDT as the positive control group showed significant neuroprotection in behavioral and infarct volume assessments, a direct comparison of VEGF and p-Akt levels between SCT and CDT groups using IHC was not performed in the current study. Future investigations could benefit from including such direct molecular comparisons to further delineate the relative mechanistic actions of SCT and established therapies like CDT.

### 2.4. RNA-Seq and Network Pharmacology Combined Analysis Reveal the Potential Therapeutic Mechanism

**Figure 5:**
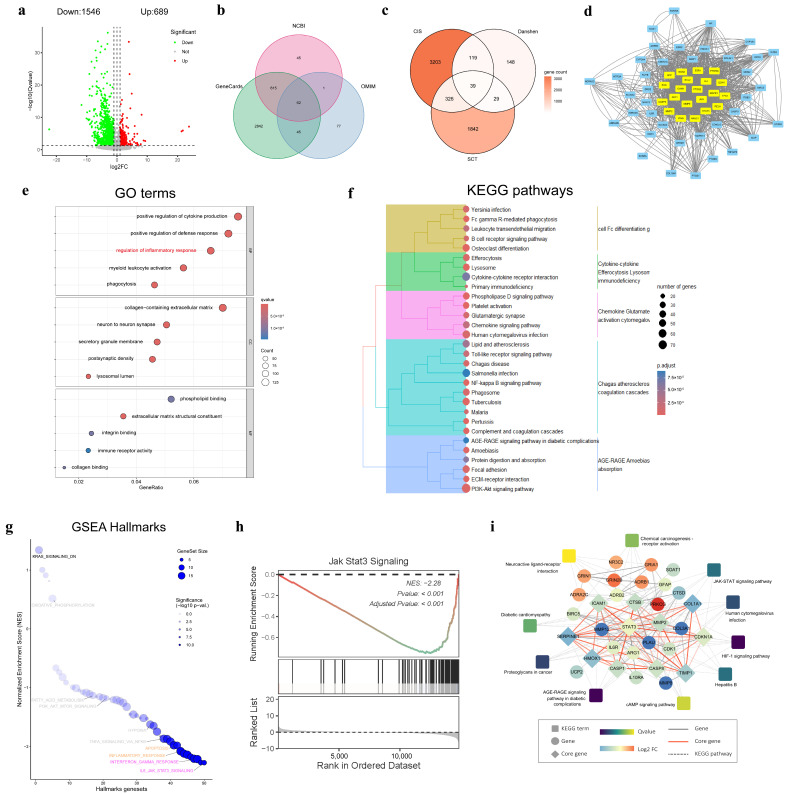
RNA-seq and network pharmacology combined analysis of rat brains between SCT-H and model groups (*n* = 3). (**a**) A total of 1,546 genes were significantly downregulated, and 689 genes were up-regulated. (**b**) Cerebral ischemia-associated genes were aggregated from authoritative databases including NCBI, GeneCards, and OMIM. (**c**) Venn diagram showed the intersected genes among CIS targets, SMB-associated targes, and DEG form RNA-seq. (**d**) The PPI network analysis revealed the interaction and identified the core genes of 39 intersecting genes. (**e**,**f**) GO ontology and KEGG pathway enrichment analysis for DEG. (**g**) Bubble plot of human hallmarks gene set enrichment analysis. (**h**) GSEA analysis curve of JAK-STAT3 signaling pathway, showing significant negative enrichment. (**i**) Target-pathway regulatory network of SCT against cerebral ischemia, with circular nodes representing core genes and square nodes representing signaling pathways.

RNA-seq analysis yielded 2,235 differentially expressed genes (DEG) between the two comparison groups, including 1,546 downregulated genes and 689 up-regulated genes (Figure 5a). To construct a target database for cerebral ischemic diseases, we integrated CIS targets databases from NCBI, GeneCards, and OMIM (Figure 5b), and accessed SMB-associated genes from TCMSP. The Venn analysis showed 39 intersected genes collected among CIS, SMB, and DEG (Figure 5c). Subsequent interaction network analysis revealed the 11 core genes with higher degrees of connectivity and scores within the network, including STAT3, MMP2, ICAM1, SERPINE1, and others (Figure 5e).

Furthermore, we conducted the Gene Ontology (GO) and Kyoto Encyclopedia of Genes and Genomes (KEGG) enrichment analysis of these DEGs to investigate the treatment mechanism of SCT. Within the GO enrichment analysis (Figure 5e), the biological process (BP) category revealed a strong association with inflammatory and immune responses. Meanwhile, the cellular component (CC) category emphasized the prominence of the collagen-containing extracellular matrix and neuron-to-neuron synapse. In the molecular function (MF) domain, phospholipid binding and actin binding were identified as significant functions. KEGG pathway analysis revealed that the AGE-RAGE signaling pathway in diabetic complications had the most significant q-value, and the PI3K-Akt signaling pathway was enriched with the highest number of gene counts (Figure 5f). Moreover, we conducted a GSEA analysis for the human disease hallmarks gene set. The results similarly showed significant enrichment in multiple pathways related to inflammation, immunity, and apoptosis (Figure 5g). Notably, the JAK-STAT3 signaling demonstrated the most significant negative enrichment, indicating it was strongly downregulated by SCT (Figure 5h).

Finally, we integrated the above analysis to construct the “target-pathway” regulatory network of SCT against cerebral ischemia (Figure 5i). This network disclosed several core genes, such as STAT3, AKT1, CTSB, and the important signaling pathways they participate in, including JAK-STAT3, HIF-1, and cAMP signaling pathways. Moreover, this regulatory network provided a systematic perspective for elucidating the potential therapeutic mechanisms of SCT.

While the antioxidant and anti-inflammatory properties of SCT are well-investigated and likely contribute to the acute neuroprotection observed in our study, the synergistic effect of the combined SCT extract might involve more complex and long-term restorative mechanisms. Further research about endogenous repair processes following brain injury using emerging technologies could expand our scope. For instance, studies utilizing metabolomics approaches have revealed that certain natural compounds, like hydroxysafflor yellow A, can promote neurogenesis and axon regeneration after experimental brain injury, highlighting these pathways as crucial targets for therapeutic intervention [40].

It is plausible that the diverse components within the SCT extract act synergistically to create a microenvironment conducive to such regenerative processes post-stroke. Furthermore, neurotrophic support, particularly via factors like Brain-Derived Neurotrophic Factor (BDNF), is critical for neuronal survival, differentiation, and synaptic plasticity. Recent studies investigating multi-component traditional medicines, such as Xuefu Zhuyu decoction, have demonstrated links between neuroprotection and the modulation of epigenetic mechanisms, specifically showing increased BDNF expression in models of brain injury [41].

To better understand the significance of the neuroprotective effects of SCT, it is useful to compare them with those reported for other plant extracts investigated under similar preclinical ischemic stroke conditions. While our primary benchmark was the clinically relevant positive control CDT, comparing SCT to other research extracts, such as *Ginkgo biloba* and *Panax notoginseng*, could provide broader scientific context. However, the direct comparisons must be made cautiously due to variations in experimental designs, particularly considering the doses administered.

## 3. Materials and Methods

### 3.1. Materials and Chemicals

The root and rhizome of SCT were collected from Linzhi, Tibet Province, China, and were identified by Professor Ge Fahuan of Sun Yat-sen University. The specimen with voucher number (SCT-220609) was deposited in Room 417, School of Pharmacy, Sun Yat-sen University. CDT was purchased from Hutchison Whampoa Guangzhou Baiyunshan Chinese Medicine Co., Ltd. (Guangzhou, China). The reference standard of FA was purchased from Shanghai Acmec Biochemical Technology Co., Ltd. (Shanghai, China). Three reference standards of SAB, DSS, and DT-I were purchased from Shanghai Yuanye Biotech Co., Ltd. (Shanghai, China). Five reference standards of RA, CA, T-IIA, T-I, and CT were purchased from National Institutes of Food and Drug Control (NIFDC, Beijing, China). The purity of all standards was higher than 98%, determined using method of normalized HPLC-UV peak areas. Methanol and acetonitrile (HPLC grade) were provided by Merck KGaA (Darmstadt, Germany). Formic acid, methanol, and 95% ethanol were obtained from Guangzhou Chemical Reagent Factory (Guangzhou, China). Carbon dioxide with a purity of 99.9% was purchased from Zhuhai Huaxin Gas Co., Ltd. (Zhuhai, China). Experimental water was purchased from Guangzhou Watson’s Food & Beverage Co., Ltd. (Guangzhou, China). Dimethyl sulfoxide (DMSO) was of HPLC grade and obtained from Sigma-Aldrich (Steinheim, Germany). Carboxymethylcellulose sodium (CMC-Na) was of analytical grade and obtained from Shanghai Yuanye Biotech Co., Ltd. (Shanghai, China). The enzyme-linked immunosorbent assay (ELISA) kits specific for TNF-α (ab236712), and IL-β (ab255730) were purchased from Abcam (Boston, MA, USA), while SOD (BC5165), and MDA (BC0025) were purchased from Solarbio (Beijing, China).

### 3.2. Extraction Optimization of Total Tanshinones and Total Phenolic Acids from SCT

The extraction optimization for total tanshinones and total phenolic acids from SCT was conducted using HPSFE and HPDE, respectively. HPSFE was performed on self-designed and commissioned technology processing of supercritical CO_2_ extraction system. The extraction vessel has a volume of 2 L, and separators I, II, and III can sequentially separate products at three levels. Two hundred grams of powered SCT was placed into a hanging basket and placed in the extraction vessel for HPSFE, with a certain volume of 95% ethanol added as the entrainment agent. After cooling the CO_2_ gas in the steel cylinder, it was compressed by a high-pressure pump to exceed the critical pressure while being heated to exceed the critical temperature. This study fixed the pressure of separators II and III at 6 and 6 MPa and the temperature of separators I, II, and III at 60, 45, and 40 °C. CO_2_ in a supercritical state dissolved the active ingredients from the material inside the extraction vessel. The extraction cycle was stopped after 1 h, and the extract was subjected to rotary evaporation below 55 °C and dried. After the extract of total tanshinones was obtained using HPSFE, the remaining residue was extracted by HPDE with the 10L/H low-temperature and high-pressure crushing and extraction equipment from ATS Engineering Ltd. It is mainly composed of three parts: pressure system, crushing system, and control system. HPDE is the use of high-pressure equipment devices to form an instantaneous pressure difference, thereby destroying the cells of the material to make it expand and break, allowing the solvent to penetrate, the release of active ingredients to increase, and other principles. A total of 30 mL of water was poured into the inlet of the device for circulation and drainage. Based on the material–liquid ratio, the remaining volume of water was poured into the SCT dregs and mixed thoroughly. Once the equipment pipeline was free of air bubbles, the mixture was poured into the inlet to circulate until the material was homogeneous. The pressure was then increased to 450 bar and maintained until the end of the extraction time. The extract was collected and centrifuged at 10,000 rpm for 10 min. The total phenolic acid extract was obtained by collecting and freeze-drying the supernatant.

Based on the group’s experience in HPSFE and HPDE, an OED was devised to optimize the extraction process for total tanshinones or total phenolic acids. Four critical factors—extraction pressure (MPa, A), extraction temperature (°C, B), entrainment agent multiplier (mL/g, C), and pressure of separator I (MPa, D)—were selected, as they significantly affect extraction of total tanshinones. Similarly, three crucial factors—material–liquid ratio (g/mL, A), extraction time (min, B), and extraction temperature (°C, C)—were chosen for their impact on extraction of total phenolic acids. An OED incorporating these factors at three levels each was designed to refine the extraction processes. The extraction yield (%) combined with the total yield (%) of four tanshinones (DT-I, CT, T-I, and T-IIA) or five phenolic acids (DSS, CA, FA, RA, and SAB) were utilized as the index to evaluate the extraction process of total tanshinones or total phenolic acids, respectively. The weight coefficients for these indices were set at a 1:1 ratio, and the results were processed using the weighted scoring method, with the total score normalized to 1. The analysis of variance tables was generated, and the *p*-values of less than 0.05 were considered to be statistically significant. Design-Expert software (version 8.0.6.1, Stat-Ease Inc., Minneapolis, USA) was used for the analysis of variance (ANOVA) of the obtained experimental data. The formulas were defined as follows:

Extraction yield (%) = Mass of extract/Mass of the material

Individual target component yield (%) = Mass of target component/Mass of the material

The total yield (%) of four tanshinones was the sum of DT-I, CT, T-I and T-IIA

The total yield (%) of four five phenolic acids was the sum of DSS, CA, FA, RA, and SAB

Combined score = (extraction yield/maximum extraction yield) × 0.5 + (total yield of active components/maximum total yield of active components) × 0.5.

### 3.3. Qualitative and Quantitative Analysis of Total Tanshinone and Total Phenolic Acid Extracts

Qualitative analysis was performed on an HPLC/MS-IT-TOF system (Shimadzu Co., Ltd., Kyoto, Japan), which has the combined strength of multi-stage (>2) fragmentation, high mass accuracy, and high resolution. Samples were accurately weighed and dissolved in 95% ethanol or water to achieve a concentration of 1 mg/mL. The sample solutions were then filtered through a 0.22 μm membrane for analysis. Chromatographic separation was achieved with an HPLC system, which consisted of a communication base module, two solvent delivery pumps, a column oven, and autosampler. Separation was achieved with a Kromasil C18 column (250 mm × 4.6 mm, 5 µm) kept at 25 °C. A 10 µL sample was injected, while the mobile phase consisted of A (0.1% formic acid in water) and B (acetonitrile). Mobile phase flow rate was set at 1.0 mL/min, with gradient elution as follows: 0–10 min, 5–15% B; 10–25 min, 15–28% B; 25–30 min, 28–55% B; 30–40 min, 55–65% B; 40–50 min, 65% B; 50–55 min, 65–70% B; 55–60 min, 70–75% B; 60–65 min, and 75–85% B. The detection wavelength was set to a full wavelength scan of 200 to 400 nm. Mass spectrometer with an electrospray ionization (ESI) source was operated in positive or negative mode. The operation parameters were set as follows: collision gas was argon (Ar), nebulizing gas (N_2_) was set at 1.5 L/min, drying gas (N_2_) pressure was 95.0 kPa, the heater block and CDL temperatures were both set at 200 °C, and detection voltage and capillary voltage were 1.80 kV and 4.5 kV/−3.5 kV, respectively. IT area vacuum and TOF area vacuum were 1.6 × 10^−2^ Pa and 1.3 × 10^−4^ Pa, respectively; ion accumulation time was 10 ms; and the ranges of *m*/*z* were set at 150–900 and 100–1200 for MS^+^ and MS^−^ scan, respectively. The collision energies were all maintained at 50%. All peak identification was performed by comparing the retention time, mass spectra, and fragmentation ions with standards and related references, and data were processed and calculated with LC/MS solution 3.7 software and Formula Predictor (Shimadzu).

The multi-indicator content determination was performed on Dionex UltiMate 3000 HPLC^+^ system (Thermo Fisher Scientific, New York, NY, USA). The SCT extracts were weighed accurately and dissolved in ethanol or water to achieve a concentration of 1 mg/mL. The solution was then filtered through a 0.45 μm membrane for HPLC analysis. Sample chromatography was performed on Kromasil C18 column (250 mm × 4.6 mm, 5 µm) adopted at 20 °C. A 10 µL sample was injected, while the mobile phase consisted of A (0.1% formic acid in water), B (methanol), and C (acetonitrile). Mobile phase flow rate was set at 1.0 mL/min, with gradient elution as follows: 0–10 min, 5% B and 5–15% C; 10–25 min, 5% B and 15–28% C; 25–30 min, 5% B and 28–55% C; 30–35 min, 5% B and 55–65% C; 35–40 min, 5–10% B and 65% C; 40–50 min, 10% B and 65% C; 50–55 min, 10% B and 65–70% C; 55–60 min, 10–5% B and 70–75% C; 60–65 min, 5–0% B and 75–85% C. The detection wavelength was set at 270 nm. The calibration curves for DSS, CA, FA, RA, SAB, DT-I, CT, T-I, and T-IIA were Y = 14.178X + 0.0078 (R^2^ = 0.9995), Y = 31.288X + 0.0005 (R^2^ = 0.9999), Y = 19.182X − 0.0146 (R^2^ = 0.9995), Y = 11.014X − 0.0853 (R^2^ = 0.9999), Y = 14.578X − 0.0164 (R^2^ = 0.9998), Y = 78.523X − 0.0116 (R^2^ = 0.9998), Y = 69.515X − 0.0002 (R^2^ = 0.9999), Y = 99.310X − 0.0191 (R^2^ = 0.9999), and Y = 80.610X − 0.0525 (R^2^ = 0.9999). The contents of total tanshinones and total phenolic acids were determined using a UV-2600 spectrophotometer (Shimadzu Co., Ltd., Kyoto, Japan) [42,43]. Total tanshinone extract (2.50 mg) was weighed and dissolved in 250 mL of 95% ethanol to prepare the sample solution of total tanshinones. Similarly, total phenolic acid extract (2.00 mg) was weighed and dissolved in 25 mL of water to prepare the sample solution for total phenolic acid. The absorbance values for total phenolic acid and total tanshinone were recorded at 286 nm and 270 nm, respectively. The calibration curves for total phenolic acid and total tanshinones were Y = 20.416X − 0.0702 (R^2^ = 0.9997) and Y = 116.740 X − 0.0055 (R^2^ = 0.9992).

### 3.4. The Neuroprotective Effect of Total Tanshinones Combined with Total Phenolic Acid Extracts of SCT Against IS

#### 3.4.1. Animal and Administration

A total of 72 Male Sprague Dawley rats (180–220 g) were purchased from Guangdong Province Medicine’s Experiment Animal Center (Guangzhou, China). The rats were randomly divided into six groups, with 12 in each group, including a sham operation group, a model group, three different dose groups of SCT (SCT-L, SCT m, and SCT-H), and a positive control group of CDT. Notably, the experimental product of SCT was combined with total tanshinone and total phenolic acid extracts under its optimal extraction conditions according to the corresponding extraction yield ratio of 1:8. All animal procedures were approved by the Institutional Animal Care and Use Committee (IACUC), Sun Yat-Sen University (approval date: 20 March 2023; approval No. SYSU-IACUC-2023–000472).

The CDT was administered according to the recommended daily oral dose for humans of 5.4 g, as indicated in the drug instructions; the rat dose of 0.54 g/kg/day was calculated through body surface area conversion, serving as the experimental dose, i.e., 1.08 g/kg/day. The doses of the combined extract from SCT were set at 0.54 g/kg/day, 1.08 g/kg/day, and 2.16 g/kg/day, for the low-, medium-, and high-dose groups, respectively.

The CDT and SCT were suspended in 0.5% CMC-Na for intragastric administration, while the administration volume was set at 1 mL/kg among all groups.

After reperfusion at day 0, the suspension was administered intragastrically once 5 min later, and once daily from day 1 to day 7 in SCT groups, while the sham group and the model group were given an equivalent volume of double-distilled water.

#### 3.4.2. Establishment of the MCAO Model

MCAO model in rats was established to monitor cerebral ischemia–reperfusion in vivo [44]. Male Sprague Dawley rats were anesthetized via intraperitoneal injection of 7% chloral hydrate (5 mL/kg), and a middle anterior incision was made in the neck to isolate and ligate the left common carotid artery and external carotid artery, as well as to separate the pterygopalatine artery. A filament was prepared proximal to the internal carotid artery, and an arterial clip was placed distally. A small incision was made at the bifurcation of the common carotid artery to insert a 4–0 nylon suture, which was advanced 17–20 mm into the internal carotid artery to enter the skull and occlude all blood flow to the middle cerebral artery (MCA). After 1 h of ischemia, the suture was withdrawn approximately 1 cm to allow reperfusion. The sham operation group underwent the same procedure without suture insertion. The wound was disinfected with povidone–iodine, and the skin was sutured.

#### 3.4.3. Evaluation of Neurological Behaviors and Function Deficits

The day of MCAO surgery was designated as day 0. To assess the neurological behavioral deficits after the surgery, the four-point scale criteria established by Zea Longa were adopted without 0 points or dead rats. The mNSS was used to evaluate the neurological function deficits after MCAO. The mNSS assessment, including four perspectives—sensory, motor, balance, and reflexes and abnormal movements—ranges from 0 to 18 points, where a higher score indicates more severe neurological impairment. Both Zea Longa method and mNSS were conducted on the animals at postoperative intervals of 1 days, 3 days, and 7 days.

#### 3.4.4. Measurement of Inflammatory Cytokines and Oxidative Stress

Seven days after the MCAO surgery, the rats were subjected to blood collection followed by serum separation. The alterations of inflammatory cytokines (TNF-α and IL-β) and oxidative stress makers (SOD and MDA) were evaluated by corresponding test assay kits according to the manufacturer’s protocols. Generally, serum was prepared on ice for subsequent testing. Following the kit instructions, serial dilutions of the standard and the working solution were prepared. The samples to be tested were then added and incubated for 30 min. After incubation, the optical density was measured at 450 nm using a microplate reader (Flex station 3, Molecular Devices, San Jose, CA, USA) within 5 min. Finally, the concentration of samples was calculated by the standard curve generated based on the concentrations of the standards.

#### 3.4.5. Determination of Brain Infarct Volume

After the blood collection for above detection, the rats were sacrificed for rapid holistic–brain extraction. The olfactory bulbs, cerebellum, and lower brainstem were removed, and the brain was then frozen for 25 min before being coronally sectioned into 3 mm thick five slices. According to our previous protocol [45], the slices were immediately immersed in a 1% solution of TTC (Sigma, USA), shielded from light, and stained at 37 °C for 20 min, with gentle agitation every 7–8 min. Upon completion of staining, the brain tissue slices were photographed, and the areas of cerebral infarction and the total brain area were quantified using Image J software (NIH, Bethesda, MD, USA, version 1.5.1). The percentage of cerebral infarction was calculated as follows: Infarct area percentage = (Infarct area/Total brain area) × 100%.

#### 3.4.6. Assessment of Cerebral Histopathology

A representative section was selected from the middle of the brain tissue slices after TTC staining. The normal hemisphere was discarded, and the infarcted hemisphere was fixed in 4% paraformaldehyde overnight. The tissue was then embedded in paraffin, and 5 μm coronal sections were prepared for HE staining. Pathological alterations in the cerebral cortex and hippocampal regions were observed under the light field using EVOS M700 imaging system (Thermo Fisher), and images were captured for documentation.

#### 3.4.7. Immunohistochemistry Staining

Portions of rat brain tissue were excised, fixed in 4% paraformaldehyde, embedded in paraffin, and sectioned. The paraffin sections were dewaxed with water, subjected to antigen retrieval, and treated to block endogenous peroxidase activity. Non-specific binding was minimized using the extract of serum, followed by overnight incubation with primary antibodies. Secondary antibodies were then applied and incubated at room temperature for 50 min. Visualization was achieved using DAB chromogen, and cell nuclei were counterstained with hematoxylin. Following dehydration and mounting, changes in the immunostaining of VEGF-α (Abcam, USA) and p-Akt (CST, Danvers, MA, USA) in the brain tissues of the sham operation group, model group, and groups treated with low and high doses of the combined extract were observed under a light microscope and documented photographically.

### 3.5. The Potential Therapeutic Mechanism of Total Tanshinones Combined with Total Phenolic Acid Extracts of SCT Against IS

#### 3.5.1. DEG Screening and Enrichment

To further investigate the neuroprotective effects of the combined extract, we conducted an RNA-seq analysis of rat brain transcriptomes of the SCT-H and model groups. After RNA was extracted and identified, we set a threshold of up-regulation (Q value < 0.05, log2 fold change > 1) and downregulation (Q value < 0.05, log2 fold change < −1) to screen DEG between the two groups. Subsequently, we subjected the DEG to a comprehensive enrichment analysis employing GO and KEGG pathways. The enrichment results were visualized using ggplot2 and clusterProlifer packages in R version 4.3.3. To investigate the disease processes associated with differential genes, gene set enrichment analysis (GSEA) of human hallmarks and visualization were performed using GseaVis package.

#### 3.5.2. Potential Treatment Targets and Interaction Network Analysis

To pinpoint the prospective treatment targets of combined extract for IS, we aggregated disease-specific targets from authoritative databases, including NCBI, GeneCards, and OMIM. Search terms such as “IS”, “cerebral infarction”, AND “Homo sapiens” were utilized, applying relevant filters where available (e.g., disease association scores in GeneCards). Concurrently, putative therapeutic genes associated with the components of SMB were retrieved from the Traditional Chinese Medicine Systems Pharmacology Database (TCMSP, https://old.tcmsp-e.com/tcmsp.php, last accessed on 30 October 2024), applying ADME-related filters (e.g., Oral Bioavailability ≥ 30% and Drug Likeness ≥ 0.18) to select active compounds and their corresponding targets. Potential therapeutic targets were then determined by intersecting the IS-related genes, the SMB putative target genes, and the DEGs using VennDiagram packages in R. The intersected genes underwent protein–protein interaction (PPI) network analysis via Cytoscape software (3.7.2) to further determine the core genes and key KEGG pathways implicated in the pathology and treatment of IS, as described in enrichment analysis.

### 3.6. Statistical Analysis

All results were statistically analyzed using GraphPad Prism 9.0 or R 4.3.3, expressed as means ± SEM unless mentioned specially. The measurement data were analyzed using one-way ANOVA with Tukey’s multiple comparisons test. Meanwhile, count data were analyzed using Kruskal–Wallis test with Dunn’s multiple comparisons test. The significance level was set at *p* value < 0.05.

## 4. Conclusions

This study pioneered the combined application of two efficient and eco-friendly extraction techniques, HPSPE and HPDE, which were used for the first time to jointly extract total tanshinones and total phenolic acids from SCT. The chemical profiles of the extracts were comprehensively characterized using HPLC, UV spectroscopy, and LC-MS for qualitative and quantitative analysis. The results revealed that HPSPE and HPDE significantly reduced the extraction time, enhanced the content of active constituents, and provided a more robust foundation for investigating SCT’s chemical composition. Furthermore, this work is the first to evaluate the neuroprotective effect of the combined extract of total tanshinones and total phenolic acids against IS. A comparative analysis demonstrated the superior efficacy of the combined extract over CDT, the conventional pharmaceutical manifestation of SMB, which is typically prepared via a traditional reflux method. Preliminary mechanistic insights into the extract’s neuroprotection were elucidated through integrated RNA-seq and network pharmacology analysis. In conclusion, the findings highlight the synergistic neuroprotective potential of SCT-derived tanshinones and phenolic acids. However, limitations persist in the thoroughness of quality assessment and mechanistic exploration of the extract and its monomeric compounds in IS. Therefore, further studies are needed to identify additional bioactive components in these extracts and delineate the precise mechanisms of action for both the extract and its key constituents.

## Figures and Tables

**Table 1:** Experimental conditions of the OED and the analysis of extreme variance for total tanshinones of SCT.

Run No.	A (MPa)	B (°C)	C (mL/g)	D (MPa)	Combined Score (Extraction Yield and Yield of Four Tanshinones Were Combined at 1:1)
1	60 (1)	60 (1)	0.5 (1)	6 (1)	0.437
2	60 (1)	55 (2)	1.5 (2)	9 (2)	0.695
3	60 (1)	50 (3)	2.5 (3)	12 (3)	0.695
4	50 (2)	60 (1)	1.5 (2)	12 (3)	0.463
5	50 (2)	55 (2)	2.5 (3)	6 (1)	1.000
6	50 (2)	50 (3)	0.5 (1)	9 (2)	0.184
7	40 (3)	60 (1)	2.5 (3)	9 (2)	0.535
8	40 (3)	55 (2)	0.5 (1)	12 (3)	0.480
9	40 (3)	50 (3)	1.5 (2)	6 (1)	0.576
^a^ k_1_	0.609	0.478	0.367	0.671	
^a^ k_2_	0.549	0.725	0.578	0.471	
^a^ k_3_	0.530	0.485	0.743	0.546	
^b^ R	0.079	0.247	0.376	0.200	
Optimal level	A_1_	B_2_	C_3_	D_1_	

^a^ ki: mean values of combined score of each factor. ^b^ R: ki,max − ki,min.

**Table 2:** Experimental conditions of OED and the analysis of extreme variance for total phenolic acids of remaining SCT residue.

Run No.	A (g/mL)	B (min)	C (°C)	Combined Score (Extraction Yield and Yield of Five Phenolic Acids Were Combined at 1:1)
1	1:20 (1)	5 (1)	4 (1)	0.712
2	1:20 (1)	15 (2)	14 (2)	0.589
3	1:20 (1)	25 (3)	24 (3)	0.662
4	1:30 (2)	5 (1)	24 (3)	0.827
5	1:30 (2)	15 (2)	4 (1)	0.664
6	1:30 (2)	25 (3)	14 (2)	0.771
7	1:40 (3)	5 (1)	14 (2)	0.632
8	1:40 (3)	15 (2)	24 (3)	0.754
9	1:40 (3)	25 (3)	4 (1)	0.911
^a^ k_1_	0.654	0.724	0.762	
^a^ k_2_	0.754	0.669	0.664	
^a^ k_3_	0.766	0.781	0.748	
^b^ R	0.112	0.112	0.098	
Optimal level	A_3_	B_3_	C_1_	

^a^ ki: mean values of combined score of each factor. ^b^ R: ki,max − ki,min.

**Table 3:** ANOVA for HPSFE of total tanshinones from SCT using OED.

Factor	Sum of Squares	Degrees of Freedom	F-Ratio	F Critical Value	Significance
A	0.010	2	0.100	4.460	-
B	0.118	2	1.174	4.460	-
C	0.213	2	2.119	4.460	-
D	0.061	2	0.607	4.460	-
Pure error	0.400	8	-	-	

F_0.05_ (2,2) = 4.460.

**Table 4:** ANOVA for HPDE of phenolic acids from remaining SCT residue using OED.

Factor	Sum of Squares	Degrees of Freedom	F-Ratio	F Critical Value	Significance
A	0.022	2	1.138	5.140	-
B	0.019	2	0.983	5.140	-
C	0.017	2	0.879	5.140	-
Pure error	0.060	6	-	-	

F_0.05_ (2,2) = 5.140.

**Table 5:** The quantitative analysis results of total phenolic acid extract.

Sample	Extraction Yield (%)	Content of Total Phenolic Acid (mg/g)	Content of Five Phenolic Acid Components (mg/g)				
			DSS	CA	FA	RA	SAB
total phenolic acid extract	26.13 ± 1.09	189.10 ± 1.47	0.74 ± 0.08	1.93 ± 0.22	0.54 ± 0.06	21.26 ± 0.29	4.54 ± 0.18

Data are means ± SD (*n* = 3).

**Table 6:** The quantitative analysis results of total tanshinone extract.

Sample	Extraction Yield (%)	Content of Total Tanshinone (mg/g)	Content of Four Tanshinone Components (mg/g)			
			DT−I	CT	T−I	T−IIA
total tanshinone extract	3.13 ± 0.42	369.43 ± 0.11	5.97 ± 0.34	39.13 ± 0.97	20.73 ± 0.27	151.27 ± 0.56

Data are means ± SD (*n* = 3).

**Table 7:** The qualitative analysis results of water-soluble components in total phenolic acid extract (positive and negative ionization modes).

No.	Ion Model	RT. (Min)	[M + H]^+^/[M − H]^−^ (*m*/*z*)		Fragment Ions (*m*/*z*)	Formula	PubChem CID	Compound Analyzed
			Calculated	Calculated				
* 1	−	8.743	395.0984	395.0987	197.054	C_9_H_10_O_5_	23693207	Danshensu
2	+	10.450	463.1235	463.1184	301.0506, 286.1035	C_22_H_22_O_11_	11016019	Diosmetin-7-o-β-D-glucopyranoside
3	+	18.790	275.1599	475.1598	313.1112, 295.0471	C_24_H_26_O_10_	-	2-[2-(4-Hydroxybenyl)-5-oxo-2,5-dihydro-3-furanyl]-5-meth-oxyphenyl beta-D-glucopyranoside
4	+	20.400	497.0867	497.0889	139.5718	C_21_H_20_O_14_	460896	3,5-Di-O-galloyquinic acid
5	+	21.028	517.1341	517.1411	325.2438	C_25_H_24_O_12_	5281780	Isochlorogenic acid B
* 6	+	22.457	181.0495	181.0521	181.0521, 163.0297	C_9_H_8_O_4_	689043	Caffeic acid
7	+	22.502	479.1548	479.1733	325.0827, 163.0440	C_23_H_26_H_11_	5273567	Calceolarioside B
8	+	24.670	447.1286	447.1388	285.1189	C_22_H_22_O_10_	5318267	Calycosin 7-o-β-D-glucoside
* 9	−	26.790	359.0772	359.0780	235.5024, 223.0207, 179.0340	C_18_H_16_O_8_	5281792	Rosmarinic acid
10	+	26.910	479.1548	479.1453	163.0443	C_23_H_26_H_11_	5273566	Calceolarioside A
11	+	27.838	315.0863	315.1038	300.0916, 243.0992, 184.0822	C_17_H_14_O_6_	13965473	3′,7-Dihydroxy-4′,6-dimethoxyisoflavone
12	+	28.293	313.1071	313.1176	253.6042	C_18_H_16_O_5_	5321620	Tanshiniol B
13	+	28.772	539.1184	539.1186	295.0967, 251.0219	C_27_H_22_O_12_	6441498	Lithospermic acid
* 14	+	28.802	741.1426 ([M + Na]^+^)	741.1454 ([M + Na]^+^)	727.1454, 519.0850	C_36_H_30_O_16_	6451084	Salvianolic acid B
15	−	28.973	717.1461	717.1425	519.795	C_36_H_30_O_16_	86278266/ 11765414	Salvianolic acid E/L
16	−	30.427	357.0616	357.0642	339.1991	C_18_H_14_O_8_	10052949/10459878	Salvianolic acid H/I
17	+	30.442	285.0758	285.0713	225.1599, 213.1902	C_16_H_12_O_5_	5280448	Calycosin
18	+	30.885	457.3829	457.3857	411.3058, 352.1478	C_30_H_48_O_3_	64945	Ursolic acid
19	+	34.310	269.0808	269.0777	226.2148	C_16_H_12_O_4_	5280378	Formononetin

* Compared with a reference standard.

**Table 8:** The qualitative analysis results of lipid-soluble components in total tanshinone extract (positive and negative ionization modes).

No.	Ion Model	RT. (Min)	[M + H]^+^/[M − H]^−^ (*m*/*z*)		Fragment Ions (*m*/*z*)	Formula	PubChem CID	Compound Analyzed
			Calculated	Calculated				
20	+	37.148	311.1278	311.1272	293.1193, 278.0720, 275.0944, 267.1271, 252.1204, 247.1112	C_19_H_18_O_4_	318797	Tanshinone IIB
21	−	38.787	295.0976	295.0944	265.8566	C_18_H_16_O_4_	149138	Danshenxinkun A
22	−	38.897	295.0976	295.1044	280.9173	C_18_H_16_O_4_	126071	Tanshinol B
23	−	39.620	313.1445	313.1484	269.2219	C_19_H_22_O_4_	389888	Neocryptotanshinone
24	+	39.635	297.1485	297.1493	269.1546, 254.1453, 253.1596, 237.0480, 223.1292	C_19_H_20_O_3_	626608	Isocryptotanshinone
25	+	39.683	337.1434	337.1404	309.1508, 263.0817	C_21_H_20_O_4_	127172	Danshenxinkun D
26	+	41.547	311.1278	311.1273	296.1552, 293.1090, 278.1037	C_19_H_18_O_4_	5318349	Hydroxytanshinone IIA
27	+	42.202	309.1121	309.1124	310.0717, 282.1034, 252.0975	C_19_H_16_O_4_	124268	Tanshinaldehyde
* 28	+	42.705	279.1016	279.1016	261.0982, 233.1394, 218.0920, 205.1570, 189.0836	C_18_H_14_O_3_	11425923	Dihydrotanshinone I
29	+	44.075	339.1227	339.1249	281.1223, 235.1182	C_20_H_18_O_5_	14610613	Methyl tanshinonate
30	+	44.325	281.1172	281.1206	253.1448, 235.1072	C_18_H_16_O_3_	5320113	Danshenxinkun B
31	−	44.438	309.1132	309.1227	290.3985	C_19_H_18_O_4_	619402	Przewaquinone A
32	+	44.972	295.1329	295.1352	295.1543, 277.1002, 249.1128, 225.0589	C_19_H_18_O_3_	626354	Isotanshinone IIA
33	−	46.080	279.0569	279.0592	251.0661	C_17_H_12_O_4_	10062187	Nortanshinone
* 34	+	48.518	297.1485	297.1476	297.1476, 282.1300, 279.1407, 254.0798, 251.1457	C_19_H_20_O_3_	160254	Cryptotanshinone
35	−	48.635	299.2017	299.1943	281.1008	C_20_H_28_O_2_	94162	Sugiol
36	−	48.718	299.1653	299.1891	284.0926, 281.0704	C_19_H_24_O_3_	10789	Tropolone
* 37	+	51.052	277.0880	277.0880	277.0880, 249.0949, 202.1097, 178.1275	C_18_H_12_O_3_	114917	Tanshinone I
38	+	55.345	279.1016	279.1016	261.0932, 233.1030, 205.1061, 205.1061, 190.0726, 189.0778	C_18_H_14_O_3_	44425164	Dihydroisotanshinone II
39	+	56.073	299.1289	301.1535, 283.2216	269.0800	C_18_H_20_O_4_	3083514	Danshenol A
* 40	+	60.282	295.1329	295.1365	295.1312, 277.1254, 249.1288, 206.1100	C_19_H_18_O_3_	164676	Tanshinone IIA
41	+	61.322	281.1536	281.1532	266.0938, 253.0989	C_19_H_20_O_2_	3082765	Dehydromiltirone
42	+	62.540	283.1693	283.1737	265.1229, 241.1234, 195.1415	C_19_H_22_O_2_	160142	Miltirone

* Compared with a reference standard.

## Data Availability

Data are contained within the article.

## Abbreviations

The following abbreviations are used in this manuscript:

T-IIA	Tanshinone IIA
RA	Rosmarinic acid.
SCT	*Salvia castanea* Diels f. *Tomentosa* Stib.
IS	Ischemic stroke.
OED	Orthogonal experimental design.
MCAO	Middle cerebral artery occlusion.
SMB	*Salvia miltiorrhiza* Bunge.
HPSFE	High-pressure supercritical fluid extraction.
HPDE	High-pressure disruption extraction.
VEGF	Vascular Endothelial Growth Factor.
p-Akt	Phosphorylation of Akt.
DSS	Danshensu sodium.
CA	Caffeic acid.
FA	Ferulic acid.
SAB	Salvianolic acid B.
DT-I	Dihydrotanshinone I.
CT	Cryptotanshinone.
T-I	Tanshinone I.
mNSS	Modified neurological severity score.
TTC	2,3,5-triphenyltetrazolium chloride.
HE	Hematoxylin and eosin.
CDTs	Compound Danshen Tablets.
DEG	Differential expression gene.
GO	Gene Ontology.
KEGG	Kyoto Encyclopedia of Genes and Genomes.
